# Stereological estimation of total cell numbers in the human cerebral and cerebellar cortex

**DOI:** 10.3389/fnhum.2014.00508

**Published:** 2014-07-15

**Authors:** Solveig Walløe, Bente Pakkenberg, Katrine Fabricius

**Affiliations:** Research Laboratory for Stereology and Neuroscience, Bispebjerg and Frederiksberg Hospitals, University of CopenhagenCopenhagen, Denmark

**Keywords:** human brain, cell numbers, development, neocortex, stereology

## Abstract

Our knowledge of the relationship between brain structure and cognitive function is still limited. Human brains and individual cortical areas vary considerably in size and shape. Studies of brain cell numbers have historically been based on biased methods, which did not always result in correct estimates and were often very time-consuming. Within the last 20–30 years, it has become possible to rely on more advanced and unbiased methods. These methods have provided us with information about fetal brain development, differences in cell numbers between men and women, the effect of age on selected brain cell populations, and disease-related changes associated with a loss of function. In that this article concerns normal brain rather than brain disorders, it focuses on normal brain development in humans and age related changes in terms of cell numbers. For comparative purposes a few examples of neocortical neuron number in other mammals are also presented.

## Introduction

The accumulation of data from quantitative stereological postmortem studies of the brain has significantly increased our knowledge about the central nervous system. Our laboratory and others have reported estimates of different types of cells in the neocortex. The number of neurons is widely considered to be a major determinant of neural function and of behavior. Brain size tends to increase with body size, but brains are relatively larger in mammals and birds than in reptiles. It has been proposed that neurons are responsible for the evolution of intelligence, in that species with larger brains and more neurons generally have a larger range and versatility of behaviors than those with smaller brains (Lefebvre et al., 2004; Sol et al., 2005). Complex cognitive capabilities are observed in a variety of animals. Complex vocal learning has been found in five distantly related groups of mammals (humans, bats, elephants, cetaceans [dolphins and whales], and pinnipeds [seals and sea lions]) (Esser, 1994; Boughman, 1998; Janik, 2000; Poole et al., 2005; Sanvito et al., 2007) and three distantly related groups of birds (parrots, songbirds, and hummingbirds) (Thorpe, 1961; Marler and Tamura, 1964; Nottebohm, 1972; Dooling et al., 1987; Baptista and Schuchmann, 1990; Gaunt et al., 1994). Self-recognition has been seen in primates and other mammals including three species of dolphins (Delfour and Marten, 2001). The use of tools is not exclusively limited to higher primates (Lefebvre et al., 2004) but has also been observed in dolphins (Delfour and Marten, 2001) and birds (Mundinger, 1980).

Much of modern neuroscience is concerned with extracting information about the content, arrangement, and connectivity of neural systems and their cell or cellular components. Often this information has been obtained from two-dimensional images of three-dimensional structures. This leads to problems in that information about the 3-D organization is lost. Previous methods involved assumptions about the size and/or shape of the sectioned profiles, e.g., that all neurons are of a certain shape of equal size and isotropically oriented (e.g., Weibel and Gomez, 1962; Rose and Rohrlich, 1987). Assumptions of this type are seldom true and can lead to systematic deviations from true estimates that cannot be corrected from the data itself.

Stereology is sampling with geometrical probes with the goal of making estimates of structural parameters. The amount of sampling can always be adjusted to a level that is efficient with respect to precision and effort (see e.g., Gundersen et al., 1988a,b; West, 2012; Mouton, 2014).

In human brain research, knowing cell numbers in selected areas can provide important information about neurodegenerative brain diseases. Modern stereological design based methods are constructed to make the quantitative descriptions of structural parameters without assumptions about shape, size, orientation, or distribution of cells. This approach has been revolutionary for the estimation of structural parameters in the brain. Recently developed stereological methods are now available for obtaining estimates of e.g., volume, surface area, particle number, and particle size. The “unbiasedness” of a stereological estimator relies heavily on proper knowledge about the structure of interest and the characteristics of the tissue. Total number can always be obtained properly using the fractionator technique or the disector/Cavalieri combination provided that all cells can potentially be identified (though not all cells will be sampled) and the sampling at all levels is carried out correctly (Gundersen, 1986; West and Gundersen, 1990). However, all estimates of volume or size are sensitive to changes in the dimension of the tissue related to histological processing; e.g., shrinkage, and must be taken into account.

In that this article is about cell numbers in the normal brain neocortex and cerebellum rather than brain disorders, it focuses on normal brain development and age related changes in humans. For comparative purposes, a few examples of neocortical neuron number in other mammals are discussed.

## Human brain development

Development of the human brain is characterized by a rapid phase of growth from conception to the second year of life. During early intrauterine life, the brain increases from about 15 gr at week 14 to about 52 gr at week 20 (247%), compared with a much smaller relative increase from 366 gr at week 36–409 gr at term (12%) (Larroche, 1977; Guihard-Costa and Larroche, 1990). The rapid initial growth of the central nervous system exceeds that of other body tissues; brain weight makes up 15–16% of body weight at 26 weeks of gestation (130/800 gr) and 12% of body weight at birth (409/3500 gr) (Guihard-Costa and Larroche, 1990), whereas in adults the brain represents only 2% of body weight (1.33/65 kg) This pattern is consistent with other mammals in which rapid growth of the brain also occurs prior to general body growth. In humans, the brain attains ~80% of its adult weight during the first 2–3 postnatal years. At the time of birth, the brain is ~30% of its adult size in humans (409/1325 gr), whereas it is 65 and 41% in related species such as the macaque monkey and chimpanzee, respectively (Prechtl, 1986; Jensen, 1996). Thus, man is a perinatal brain developer, with the maximal brain growth extending from the last half of gestation into the second and third year of life (Dobbing and Sands, 1973). The formation of the human neocortex is critically important because disturbances in the formation of cells can cause congenital brain disorders and result in adverse effects on brain morphology (i.e., abnormal proliferation, differentiation, and/or migration of cells), including quantitative changes such as decreases in the number of neurons at a specific location of their final destination.

### Delineations of developmental zones

The neocortex forms within the dorsal walls of the telencephalic vesicles, where neurogenesis is initiated in an epithelial sheet of dividing progenitor cells (Kornack, 2000). Before the onset of neurogenesis, these progenitors divide symmetrically to make more of themselves, establishing a progenitor pool. Once neurogenesis begins, some progenitor cells switch to asymmetric division to produce the first postmitotic neurons (Rakic, 1972; Kornack, 2000). The post mitotic neuron will leave the ventricular zone (VZ) by post mitotic migration along radial glial cell fibers and settle just outside the VZ, where they form the preplate and later the cortical plate (CP). After the onset of neurogenesis, dividing cells start to appear at the basal border of the VZ. Accumulation of these intermediate or “basal” progenitors creates a distinct new compartment above the VZ, which was named the subventricular zone (SVZ) (Byron et al., 2008). Originally the SVZ was thought to generate mainly glia, but later it was shown that early SVZ progenitors are largely neurogenic and many cortical neurons originate from the SVZ in mice, monkeys, and humans (Letinic et al., 2002; Brazel et al., 2003; Rakic, 2003; Zecevic et al., 2005). As the CP grows, it differentiates into an inner subplate (SP) and an outer marginal zone (MZ) that becomes the agranular layer I of the mature cerebral cortex (Rakic, 1972; Kornack, 2000). The initial post mitotic neurons settle within deep cortical layers, whereas subsequent neurons settle in more superficial cortical layers, thereby establishing the layers designated layers VI-II. As neurogenesis stops around embryonic day 125, symmetric cell division replaces the progenitor cell pool in the VZ with undifferentiated post mitotic neurons (Kornack, 2000). Subsequently, cell division stops in the VZ and post mitotic neurons are eventually replaced with ependymal cells (Kornack, 2000). Thus, due to the inside-out development of the neocortex, with its prominent proliferative zone around the VZ, it is easy to establish and delineate the developing intermediate zone (IZ). At this stage, the region consists of migrating cells and ingrowing axons and will later develop into white matter. Therefore, the cell density in the IZ is less than the VZ and is separated from the SP by the presence of horizontal axons at the SP border. The SP disappears by the sixth postnatal month (Kostovic and Judas, 1995).

### First period: 13–20 weeks of gestation

During this period, the CP/MZ is characterized by a rapid exponential growth in cell number, that reaches 5.87 × 10^9^ cells at 20 weeks of gestation (Samuelsen et al., 2003). This finding is in agreement with the qualitative observation of the secondary consolidation of the CP during 16–18 weeks of gestation (Kostovic and Judas, 1995). From week 13 to 20 of gestation, the SP includes three different developmental stages: SP *formation* at 13–15 weeks, the *expansive* phase at 16–18 weeks, and the *stationary* phase at 19–20 weeks. During these periods, neurons mature in the SP and form connections with ingrowing afferents from the brainstem, basal forebrain, thalamus, and ipsilateral and contralateral cortices (Kostovic and Judas, 1995).

### Second period: from mid-gestation to term

**Figure 1:**
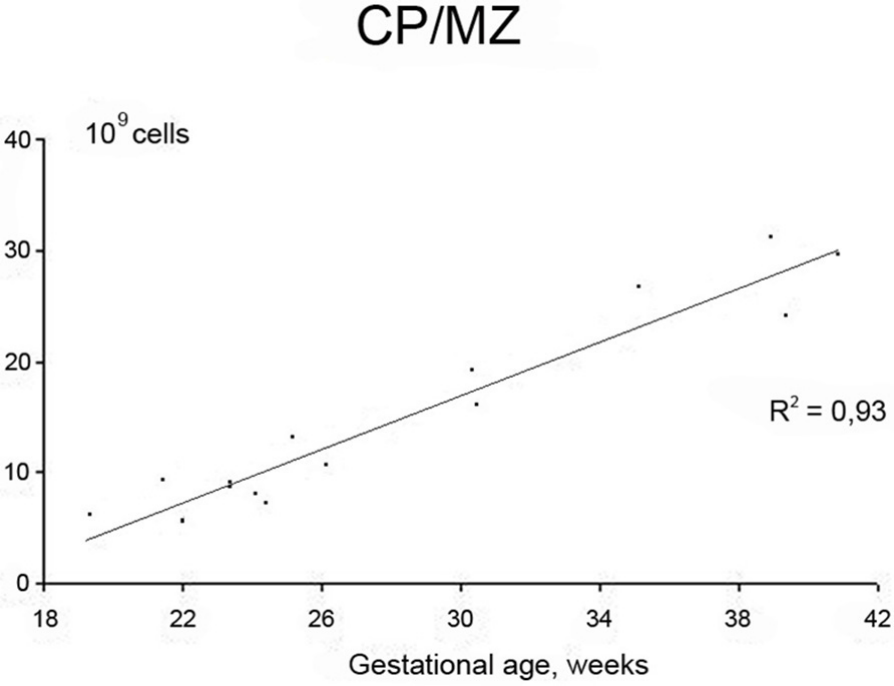
**Increase in total cell number in the cortical plate (CP)/marginal zone (MZ) in 15 normal human fetuses between 18 and 42 weeks of gestation**. Modified from Samuelsen et al. (2003).

**Figure 2:**
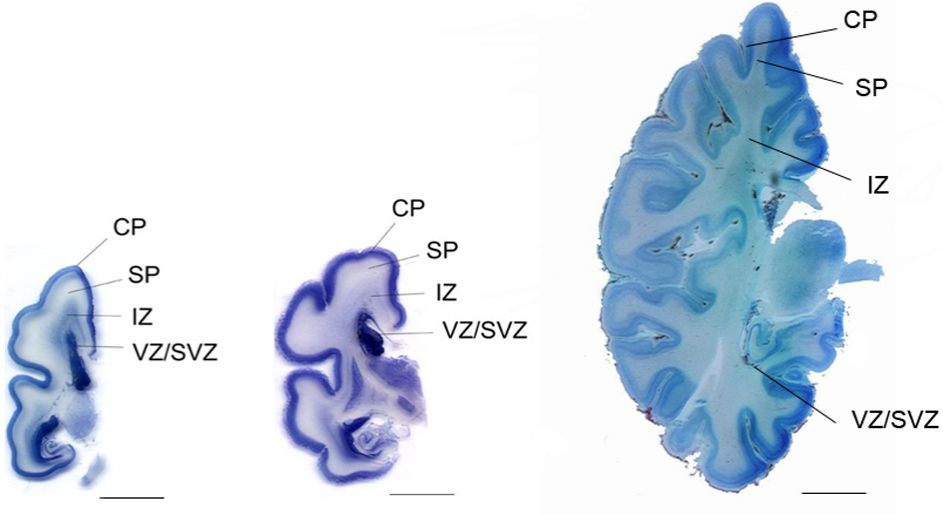
**Coronal section of a 24-week (left), 25-week (middle), and 40-week-old human fetus at the level of the basal ganglia**. CP, cortical plate; SP, subplate; IZ, intermediate zone; VZ/SVZ, ventricular zone/subventricular zone. Scale bar = 1 cm.

Neurogenesis in humans was generally assumed to be complete at mid-gestation (Rakic, 1972, 1988). However, fetal neurogenesis is not complete at 20 weeks of gestation and may in fact continue for another few weeks. This is supported by the data from Samuelsen et al. (2003) where the total cell numbers in the prospective neocortex increases during the second period, from 7.02 × 10^9^ cells at 22 weeks of gestation to 29.4 × 10^9^ cells at term. Furthermore, a subpopulation of GABAergic neurons migrates from the ganglionic eminence of the ventral forebrain to the dorsal forebrain at around 20 weeks of gestation. These clones of GABAergic neurons appear in the human fetal cerebral cortex (Letinic et al., 2002). The SP is composed of ~2.0 × 10^9^ cells at 22 weeks of gestation, and reaches a maximum of around 3.6 × 10^9^ cells at 35 weeks of gestation. Thus, in terms of total cell number, the SP is in a dynamic phase with regard to neuron number from 22 to 35 weeks of gestation. An initial growth in total cell number takes place between 20 and 35 weeks, after which the total cell number declines. The SP is thus a transitional zone that begins to disappear after 35 weeks of gestation. At term, however, the SP still contains about 3 × 10^9^ cells, suggesting that cellular interactions between immature neurons and incoming fibers in this zone could be influenced by early postnatal events (Samuelsen et al., 2003). Figure 1 shows the increase in total cell number in the CP/MZ in 15 normal human fetuses between 18 and 42 weeks of gestation (modified from Samuelsen et al., 2003). To illustrate the different developmental zones, coronal sections of a 24-, 25-, and 40-week-old human fetus at the level of the basal ganglia are shown in Figure 2. At term the total neocortical numbers are 20 × 10^9^ neurons and 5.5 × 10^9^ glial cells (Larsen et al., 2006). In sum, there is a dramatic increase in the cell numbers from 13 to 40 weeks of gestation. These numbers can serve as a normative reference in the analysis of normal fetal development. At term, the neocortical neurons are in large parts formed while the glial cell numbers continue to increase well into the first years of life.

## Estimates of the total number of neocortical neurons in the adult human brain

It is well established that males, on average, have brains that are ~150 grams larger than those of females. However, the relationship between total neuron number and brain size was not known until total number of neurons were estimated in 94 normal Danish individuals between 18 and 93 years of age using stereological methods (Pakkenberg and Gundersen, 1997). It was found that the total number of neocortical neurons in females was ~19 × 10^9^, whereas in males it was ~23 × 10^9^, which accounts for a sex-related difference of 16%. However, this difference should be seen in view of the fact, that the total number of neocortical neurons varies among individuals by more than a factor of two, with a range of 118% (14.7–32.0 × 10^9^ neurons), i.e., there is considerable overlap between men and women.

**Figure 3:**
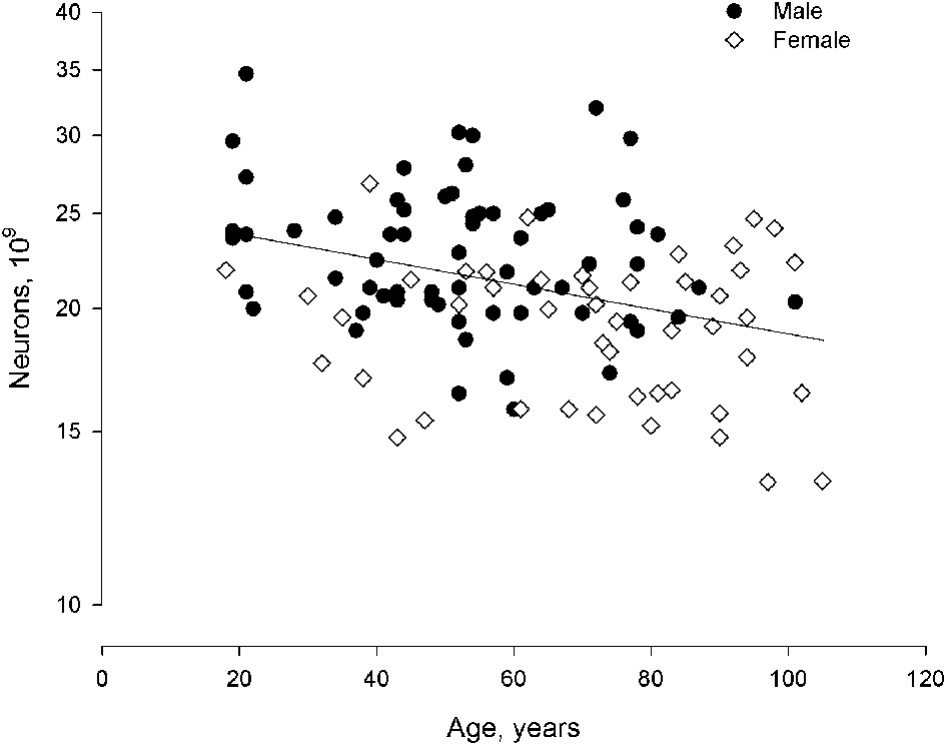
**Total neuron number (non-equidistant logarithmic scale) as a function of age (linear scale: 18–105 years) in human males and females**. In a larger material it would be expected that the curve flattens as we grow very old.

With age, reductions occurred in neocortical volume, surface area, white matter, archicortex volume, and brain weight. These occurred concomitant with a large increase in the ventricular system. There were no changes in gray matter volume or neocortical thickness. Average volume and numerical neuronal density of the four lobes of the neocortex are shown in Table 1 (modified from Pakkenberg and Gundersen, 1997). After correcting for sex and age differences, neocortical neuron number is the dominant factor in determining the size of other brain structures. Neuronal density does not vary as a function of either sex or age (Table 1). Sex and age are the main determinants of the total neocortical neuron number, whereas body size, *per se*, has no influence. The change in total number of neocortical neurons from age 18 to 93 years is 9.5%, resulting in an average “loss” of about 85,000 neurons per day or ~1 per second. This age-dependent neuronal decrease is equivalent for both sexes. Finally, Fabricius et al. (2013) found that the total neocortical neuron number in individuals between 94 and 105 years of age (seven females, one male) is the same in very old females compared with younger women, group 1: 65–75 years (*n* = 8), and group 2: 76–85 years (*n* = 8). Although the total neuron number was the same in the 3 groups, the variance of the estimates increased substantially with age, indicating that apart from their high age, the variance in cell numbers suggests a relatively inhomogeneous sample. The estimates of neocortical neuron number from 18 to 105 years of age are shown in Figure 3. It was concluded, that either these very old subjects were born with more neurons than their peers, or some individuals lose fewer neurons over time as they age. Having a high neocortical neuron number may give a biological advantage that could be related to longevity.

**Table 1:** **Geometric mean and CV (in parentheses) for volume, and numerical density (N_v_) in the four subdivisions of the human neocortex**.

**Region**	**Sex**	**Volume, cm^3^**	**N_v_ × 10^6^/cm^3^**
Frontal	M	213 (0.18)	36.7 (0.18)
	F	184 (0.20)	35.9 (0.15)
Temporal	M	120 (0.17)	59.8 (0.17)
	F	102 (0.20)	51.0 (0.20)
Parietal	M	117 (0.20)	47.3 (0.19)
	F	100 (0.22)	45.2 (0.19)
Occipital	M	64 (0.29)	66.9 (0.19)
	F	51 (0.23)	70.9 (0.20)

## Total neocortical glial cell number in the normal adult brain

**Figure 4:**
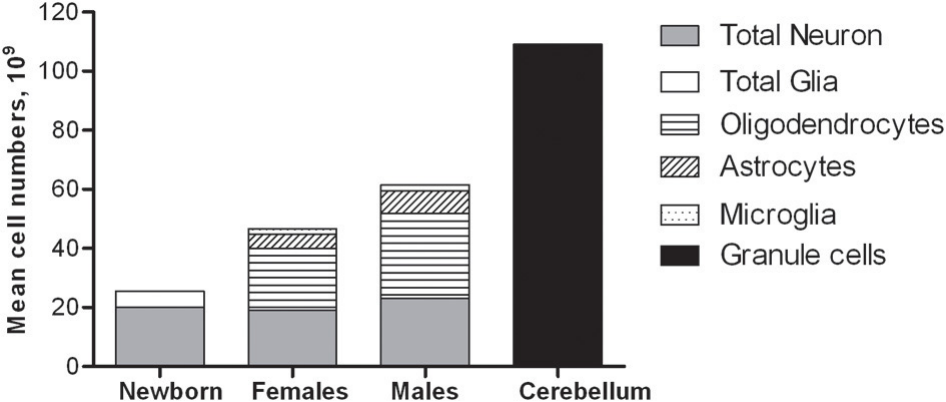
**Mean total neuron and glial cell number in the human neocortex and cerebellum**.

The total number of neocortical glial cells was estimated (Pelvig et al., 2008) in some of the same brains used for estimating neocortical neuron number (Pakkenberg and Gundersen, 1997). Pelvig et al. (2008), Table 2 included the neocortices of 13 Caucasian males and 18 females (range: 18–93 years of age), and differentiated between astrocytes, oligodendrocytes, microglia, and neurons in each of the four neocortical lobes (Pelvig et al., 2008). The mean total numbers were ~21.0 × 10^9^ oligodendrocytes in females and 28.8 × 10^9^ oligodendrocytes in males, 4.8 × 10^9^ astrocytes in females and 7.8 × 10^9^ astrocytes in males, and 1.8 × 10^9^ microglia in females and 2.0 × 10^9^ microglia in males. The total glial cell numbers were 27.9 × 10^9^ in females and 38.9 × 10^9^ in males, so males have ~28% more neocortical glial cell number than females. In brains of equal size, there were no sex-related differences in neuron or glial numbers (Pelvig et al., 2008). The number of oligodendrocytes showed a significant 27% decrease during adult life and a strong correlation to the total number of neurons. The total astrocyte number is constant through life. The mean total neuron and glial cell numbers in the neocortex and cerebellum is shown in Figure 4. Finally, brains of equal size have the same number of glial cells. The different subpopulations of glial cells behave differently as a function of age. Generally, the number of glia cells has a higher biological variance than the number of neurons.

**Table 2:** **Summary of the cell number presented in this paper**.

**Region**	**Species**	**Brain weight (g)**	**Cell type**	**Total number of cells**	**Method**	**References**
Neocortext	Human, *Homo sapien*, (m)	m: 1400; f: 1250	Neuron	23 × 10^9^	Optical disector × Cavalieri	Pakkenberg and Gundersen, 1997
–	– (f)		–	19 × 10^9^	–	–
–	– at term		–	20 × 10^9^	Optical fractionator	Larsen et al., 2006
–	– at term		Glia	5.5 × 10^9^	–	–
–	– (m)		–	38.9 × 10^9^	Optical disector × Cavalieri	Pelvig et al., 2008
–	– (f)		–	27.9 × 10^9^	–	–
–	– (m)		Oligodendrocyte	28.8 × 10^9^	–	–
–	– (f)		–	21 × 10^9^	–	–
–	– (m)		Astrocyte	7.8 × 10^9^	–	–
–	– (f)		–	4.8 × 10^9^	–	–
–	– (m)		Microglia	2.0 × 10^9^	–	–
–	– (f)		–	1.8 × 10^9^	–	–
–^**^	– 22 weeks gestation		Total cells	7.02 × 10^9^	Optical fractionator	Samuelsen et al., 2003
–^**^	– at term		–	29.4 × 10^9^	–	–
Frontal cortex	– (m)		Neuron	7.8 × 10^9^	Optical disector × Cavalieri	Pakkenberg and Gundersen, 1997
–	– (f)		–	6.6 × 10^9^	–	–
Frontal cortex	–		–	7.8 × 10^9^	–	Gredal et al., 2000
Temporal cortex	– (m)		–	4.9 × 10^9^	–	Pakkenberg and Gundersen, 1997
–	– (f)		–	4.3 × 10^9^	–	–
Parietal cortex	– (m)		–	5.5 × 10^9^	–	–
–	– (f)		–	4.5 × 10^9^	–	–
Occipital cortex	– (m)		–	4.2 × 10^9^	–	–
–	– (f)		–	3.6 × 10^9^	–	–
Motor cortex	–		–	1.3 × 10^9^	–	Gredal et al., 2000
Cerebellum	– (m)		Purkinje	28 × 10^6^	–	Andersen et al., 2003
–	– 1 month		–	12.1 × 10^6^	Optical fractionator	Kiessling et al., 2014
–	– 11 months		–	13.9 × 10^6^	–	–
–	– (m)		Granule	109 × 10^9^	Optical disector × Cavalieri	Andersen et al., 2003
–	– 1 month		–	5.9 × 10^9^	Optical fractionator	Kiessling et al., 2014
–	– 11 months		–	37.6 × 10^9^	–	–
CP/MZ	– 20 weeks gestation		Total cells	5.87 × 10^9^	Optical fractionator	Samuelsen et al., 2003
SP	– 22 weeks gestation		–	2 × 10^9^	–	–
–	– 35 weeks gestation		–	3.6 × 10^9^	–	–
–	– at term		–	3.0 × 10^9^	–	–
**OTHER MAMMALS**						
Neocortex	Rhesus macaque, *Macaca mulatta*	80–100	Neuron	2.8 × 10^9^	Optical fractionator	Christensen et al., 2007
–	Minke whale, *Balaenoptera acutorostrata*	2140	–	13 × 10^9^	–	Eriksen and Pakkenberg, 2007
–	Harbor porpoise, *Phocoena phocoena*	413	–	15 × 10^9^	Optical disector × Cavalieri	Walløe et al., 2010
–	Harp seal, *Pagophillus groenlandicus*	215	Neuron	6.0 × 10^9^	Optical disector × Cavalieri	Walløe et al., 2010
–	Brown rat, *Rattus norvegicus*	156	–	20 × 10^6^	Optical disector × Cavalieri	Korbo et al., 1990
–	Gottingen minipig, *Sus scrofa domestica*	79	–	325 × 10^6^	Optical fractionator	Jelsing et al., 2006
–	Domestic pig, *Sus domesticus*	134	–	430 × 10^6^	–	–

** *Prospective neocortex; m, male; f, female; –, the same as the previous*.

## The human cerebellum

Applying stereological methods for cell counting to postmortem cerebella from 14 children who died between the first postnatal day and 11 months of age, Kiessling et al. (2014) found a statistically significant age-related increase in the total number of granule cells from 5.9 × 10^9^ at 1 month to 37.6 × 10^9^ at month 10/11 per cerebellar half. They did not find a difference in the total number of Purkinje cells (12.1 × 10^6^ at month 1 vs. 13.9 × 10^6^ at month 10/11 per cerebellar half). The number of granule cells per Purkinje cell in the human cerebellum increases from 485 at postnatal month 1 to 2700 at month 10/11. They concluded that these data indicate that the human cerebellum have a much higher functional plasticity during the first year of life than previously thought.

Unbiased stereological methods haves also been applied to cerebella from 19 normal Caucasian males aged 19–84 years (Andersen et al., 2003). Total volume of the cerebellar cortex and white matter, cerebellar surface area, total Purkinje and granule cell number, and the distribution of the mean volume of Purkinje cells and their nuclei were estimated in the anterior lobe, the posterior lobe, the flocculonodular lobe, and vermis. Across age, it was found that global white matter decreased by 26%, the mean volume of Purkinje cell bodies decreased by 33%, and there was no decrease in volume of Purkinje cell nuclei. Furthermore, a 16% total cerebellar volume loss was seen without a concomitant neuron loss. No global Purkinje or granule cell loss was found with age, with total Purkinje cell number being 28 × 10^6^ and total granule cell number being 109 × 10^9^. However, with age a significant loss of both Purkinje and granule cells were seen in the anterior lobe. Furthermore, a 30% loss of volume, mostly due to cortical volume loss, was observed in the anterior lobe (Andersen et al., 2003).

In sum, ~85% of the cerebellar granule cells are generated postnatally in humans. Most regions in the human cerebellum show only minor age related morphological changes. The anterior lobe is a major exception, with a 40% reduction in the total number of granule and Purkinje cells and a 28% loss of cortical volume, mainly due to reductions in the granule cell layer. The anterior lobe is functionally related to the spinal cord and is mainly concerned with posture, muscle tone, and gait, that is, motor functions, which are often affected by aging. Globally, cerebellar white matter volume is significantly reduced with age and is affected to almost the same degree (26%) as white matter in the cerebral hemispheres (28%).

## Total cell number in the cerebrum

Interest has been shown to the total number of cells in the normal brain (Herculano-Houzel and Lent, 2005; Herculano-Houzel et al., 2007; Herculano-Houzel, 2011). Using a non-stereological method, the isotrophic fractionator, Azevedo et al. (2009) found the total number of cells in four brains from 50-, 51-, 54-, and 71- year-old males, deceased from non-neurological causes and without cognitive impairment to have on average 86.1 ± 8.1 billion NeuN-positive cells (“neurons”) and 84.6 ± 9.8 billion NeuN-negative (“non-neuronal”) cells. Using a fractionator sampling scheme and optical disector probes (Gundersen et al., 1988b; West et al., 1991; West, 1993), we estimated the total number of neurons and glial cells in the cerebrum of four normal Danish subjects. Subjects were between 62 and 71 years of age (three males, one female) and had been included in another study. The results, which are shown in Table 3, are within the normal range though in the lower end. This could be due to age and a small sample size. The results by Azevedo et al. (2009), excluding cerebellum, is within normal numbers. The isotrophic fractionator method can thus offer reliable total numbers when total numbers are of interest, provided the cells can be identified correctly by a staining method. Using stereological methods, the anatomical brain structures, and the specific regions are preserved and total numbers can be combined with estimates of regional brain volumes, surfaces, fiber length, cell distances, pathological markers etc. Further, the brain can be used repeatedly in new studies and with new scientific issues, which is of special importance in human brain research, where the tissue is so valuable. In sum, the biological variance in the number of cells from brain to brain, in both selected brain regions and the entire hemisphere, is high. This is so, even for brains of approximately the same age. It is thus difficult to give an average for humans because of rather large differences between males and females (~15%) and with age (~10%) (Pakkenberg and Gundersen, 1997).

**Table 3:** Details of the stereological procedure for determining total number of cells in the human cerebrum^*^ using the optical fractionator method.

**Case no**.	**∑s**	**ssf^−1^**	**asf^−1^**	**h(μm)**	***t*(μm)**	**hsf^−1^**	**∑CF**	**∑n**	**∑g**	**CE**	**Total glia**	**Total neuron**	**Total no. cells**
1	14	0.003	2 × 10^−5^	15	44.9	0.33	563	272	877	0.05	77 × 10^9^	24 × 10^9^	101 × 10^9^
2	20	0.005	2 × 10^−5^	15	39.2	0.38	409	325	1274	0.04	65 × 10^9^	17 × 10^9^	82 × 10^9^
3	19	0.005	2 × 10^−5^	15	39.7	0.38	777	347	1282	0.04	67 × 10^9^	18 × 10^9^	85 × 10^9^
4	20	0.005	2 × 10^−5^	15	37.9	0.39	663	325	1353	0.05	67 × 10^9^	16 × 10^9^	83 × 10^9^
Mean	18	0.0045	2 × 10^−5^	15	40.4	0.37	603	317	1197	0.045	69 × 10^9^	19 × 10^9^	88 × 10^9^

*ssf-1, reciprocal value of the section sampling fraction; asf-1, reciprocal value of the area sampling fraction; h, height of the counting frame; t, section thickness; hsf1, reciprocal value of the thickness sampling fraction; **∑** CF, number of unbiased counting frames used; **∑** n, number of neurons counted; **∑** g, number of glia cells counted; CE, coefficients of error of the estimates as in Gundersen et al. (1999)*.

* *Cerebrum includes the whole hemisphere, e.g., cortex, white matter, and central gray*.

## Other mammals

**Figure 5:**
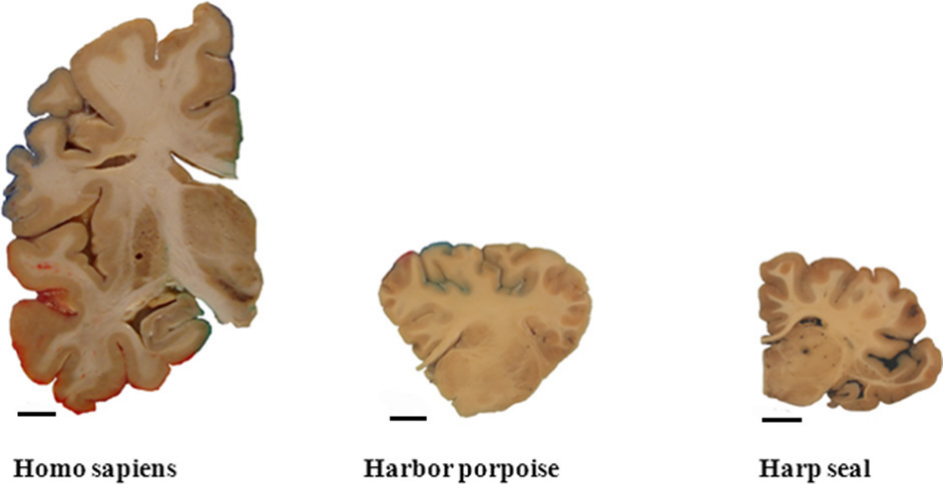
**A prominent aspect of the human brain compared with brains of other species is its large size**. Coronal sections through a hemisphere of an adult human, an adult harbor porpoise, and an adult harp seal brain (Part of the picture is with courtesy of Walløe et al., 2010). Scale bar = 1 cm.

A prominent aspect of the human brain compared with brains of other species is its large size and high number of neurons (Figure 5). For comparison we have included estimates of the total number of neocortical neurons from other mammals, using similar modern stereological methods. The numbers are shown in Table 2. Relatively speaking, the human brain is the biggest primate brain, being three times larger than the brain of great apes. Large mammals such as elephants and whales have larger brains than humans, but only because brain weight is linearly correlated with body weight in mammals (Armstrong, 1990). Primate neocortical size increases predictably, as overall brain size increases, and the human neocortex follows this scaling rule. The prefrontal cortex makes up nearly 30% of the entire neocortex in humans, 17% in chimpanzees, and about 10% in small primates such as the M. maurus (11%) or marmoset (9%) (Brodmann, 1912). A chimpanzee-to-human brain warping study based on a limited set of homologies showed selective expansion of human prefrontal and lateral temporal association cortices (Avant et al., 2006). The human frontal cortex displays a higher ratio of glia to neurons than in other anthropoid primates. Sherwood et al. (2006) estimated cell densities using optical disectors in layer II/III of dorsolateral prefrontal cortex (area 9L) in the left hemisphere of brain samples representing 18 anthropoid primate species. They found that the human frontal cortex displays a higher ratio of glia to neurons than in other anthropoid primates. This relative increase in glia conformed to allometric scaling expectations, when taking the large increase of the human brain into consideration. They suggested that the relatively greater numbers of glia in the human neocortex relate to the energetic costs of maintaining larger dendritic arbors and long-range projecting axons in the context of a large brain. Comparing the spatial organization of neurons in the cortex of humans and great apes, Semendeferi et al. (2011) found differences in the frontal pole between humans and apes. The so-called horizontal spacing distance between neurons was 30% larger in humans compared to other species including bonobo, chimpanzee, gorilla, orangutan, and gibbon. Thus in many ways the prefrontal cortex separates humans from other mammals. These results are supported by estimates of neuron numbers. With an average bilateral total neuron number of ~7.8 × 10^9^ in the frontal cortex and ~1.3 × 10^9^ in the motor cortex (Gredal et al., 2000), average neocortical neuron number in the human prefrontal cortex is thus ~6.5 × 10^9^ or close to one-third of the number of all neocortical neurons. Compared with other living primate species, relatively more of the human cerebral cortex is dedicated to conceptual and other forms of higher-order cognitive processing as opposed to perceptual processing (Rilling, 2013).

## Discussion

Our knowledge about the relationship between brain structure and cognitive function is limited by the considerable variability of the brain in size and shape, including the size of individual cortical areas, and the fact that most historical data were collected by potentially biased methods. Within the last 20–30 years, it has become possible to obtain more reliable data with unbiased stereological methods. These methods have provided us with new information about, for example, differences in cell number between men and women, the effect of age on selected brain cell populations, and disease-related changes associated with a loss of function (Pakkenberg and Gundersen, 1997; Andersen et al., 2003; Fabricius et al., 2007; Karlsen and Pakkenberg, 2011; Fabricius et al., 2013). Normal neocortical neuron numbers were found in chronic alcoholics (Jensen and Pakkenberg, 1993; Fabricius et al., 2007, 2013). This finding can have a major impact on the treatment of chronic alcoholism in that brain cells are not lost even after years of excessive alcohol consumption. The patients can therefore expect that brain function can be restored to normal if the abuse is terminated, even in subjects with a long history of alcohol abuse. Other studies provide notable results, including the observation that neocortical neuron number is normal in severely demented individuals with Alzheimer's diseases (Regeur et al., 1994; Pelvig et al., 2003), whereas total neocortical neuron number is significantly reduced in individuals dying from Acquired Immune Deficiency Syndrome (AIDS) without dementia (Oster et al., 1995). The stereological methods are also highly relevant to comparative neurology where such data can be used to illuminate the human brain's place in nature. Future research will undoubtedly provide additional knowledge about the human brain, its complexity, and changes caused by disease.

### Conflict of interest statement

The authors declare that the research was conducted in the absence of any commercial or financial relationships that could be construed as a potential conflict of interest.
